# Protein Tyrosine Phosphatase PTP1B Is Involved in Hippocampal Synapse Formation and Learning

**DOI:** 10.1371/journal.pone.0041536

**Published:** 2012-07-23

**Authors:** Federico Fuentes, Derek Zimmer, Marybless Atienza, Jamie Schottenfeld, Ian Penkala, Tracy Bale, Kendra K. Bence, Carlos O. Arregui

**Affiliations:** 1 Instituto de Investigaciones Biotecnológicas, Universidad de San Martín/CONICET, San Martín, Buenos Aires, Argentina; 2 Department of Animal Biology, School of Veterinary Medicine, University of Pennsylvania, Philadelphia, Pennsylvania, United States of America; University of Nebraska Medical Center, United States of America

## Abstract

ER-bound PTP1B is expressed in hippocampal neurons, and accumulates among neurite contacts. PTP1B dephosphorylates ß-catenin in N-cadherin complexes ensuring cell-cell adhesion. Here we show that endogenous PTP1B, as well as expressed GFP-PTP1B, are present in dendritic spines of hippocampal neurons in culture. GFP-PTP1B overexpression does not affect filopodial density or length. In contrast, impairment of PTP1B function or genetic PTP1B-deficiency leads to increased filopodia-like dendritic spines and a reduction in mushroom-like spines, while spine density is unaffected. These morphological alterations are accompanied by a disorganization of pre- and post-synapses, as judged by decreased clustering of synapsin-1 and PSD-95, and suggest a dynamic synaptic phenotype. Notably, levels of ß-catenin-Tyr-654 phosphorylation increased ∼5-fold in the hippocampus of adult PTP1B^−/−^ (KO) mice compared to wild type (WT) mice and this was accompanied by a reduction in the amount of ß-catenin associated with N-cadherin. To determine whether PTP1B-deficiency alters learning and memory, we generated mice lacking PTP1B in the hippocampus and cortex (PTP1B^fl/fl^–Emx1-Cre). PTP1B^fl/fl^–Emx1-Cre mice displayed improved performance in the Barnes maze (decreased time to find and enter target hole), utilized a more efficient strategy (cued), and had better recall compared to WT controls. Our results implicate PTP1B in structural plasticity within the hippocampus, likely through modulation of N-cadherin function by ensuring dephosphorylation of ß-catenin on Tyr-654. Disruption of hippocampal PTP1B function or expression leads to elongation of dendritic filopodia and improved learning and memory, demonstrating an exciting novel role for this phosphatase.

## Introduction

The hippocampus has been implicated in memory formation and learning; both of these processes are accompanied by specific modifications in the structure and function of the synapse [1]–[5]. The role of N-cadherin and the associated catenins in synapses has been well documented both *in vitro* and *in vivo* [6]–[8]. For example, impairment of N-cadherin function inhibits the induction of LTP and structural plasticity [8]–[12]. Expression of a dominant negative N-cadherin or deletion of α-catenin in cultured hippocampal neurons leads to aberrant dendritic spines [13], [14]. *In vivo* conditional deletion of ß-catenin in newborn neurons of postnatal dentate gyrus impairs the formation of branched dendrites [15].

N-cadherin function relies on dynamic interactions with the actin cytoskeleton, in a process mediated by catenins and regulated by tyrosine phosphorylation [16]. Binding of ß-catenin to the cytoplasmic domain of N-cadherin is negatively regulated by the phosphorylation of ß-catenin-Tyr-654 [17]. The role of N-cadherin in memory formation and retrieval has recently been evaluated *in vivo*. N-cadherin levels quickly rise in the hippocampus after fear conditioning, and then decrease slowly [18]. Disruption of hippocampal N-cadherin function impaired the consolidation but not the retrieval of contextual fear memory [18]. Similarly, another study showed that conditional deletion of ß-catenin in the amygdala impairs consolidation but not acquisition of memory [19]. This work also showed that total ß-catenin protein levels in the basolateral amygdala do not change after fear conditioning; however, phosphorylation of the ß-catenin-Tyr-654 residue and subsequent N-cadherin/ß-catenin interactions are dynamically regulated [19] highlighting the potential importance of Tyr-654 phosphorylation in learning.

PTP1B is an ER-anchored enzyme with the catalytic domain facing the cytosol [20]. PTP1B dephosphorylates ß-catenin, and positively regulates N-cadherin-mediated adhesion [21]–[23]. We recently have shown that the dynamic distribution of ER-bound PTP1B in hippocampal neurons depends on microtubules [24]; microtubules transiently invade dendritic spines, and neuronal activity enhances this process [25], [26]. A specific role for PTP1B within hippocampal dendritic spines, however, has not been explored.

Here we show that PTP1B is dynamically present in dendritic spines. Impairing PTP1B function by expression of a dominant negative mutant or by genetic deletion reveals that it is required for spine maturation and normal synapse formation. *In vivo*, PTP1B regulates the dephosphorylation of ß-catenin on Tyr-654 in the hippocampus, and contributes to the formation of stable N-cadherin/ß-catenin complexes. Finally, mice with PTP1B-deficiency specifically in the hippocampus and cortex (PTP1B^fl/fl^-Emx1-Cre) display improved performance compared to WT controls in the Barnes maze (a learning paradigm for mice). Thus, we present biochemical, structural and behavioral data suggesting an exciting role for PTP1B in hippocampal function and structural plasticity.

## Results

### PTP1B Localizes Transiently in the Post-synaptic Compartment

**Figure 1:**
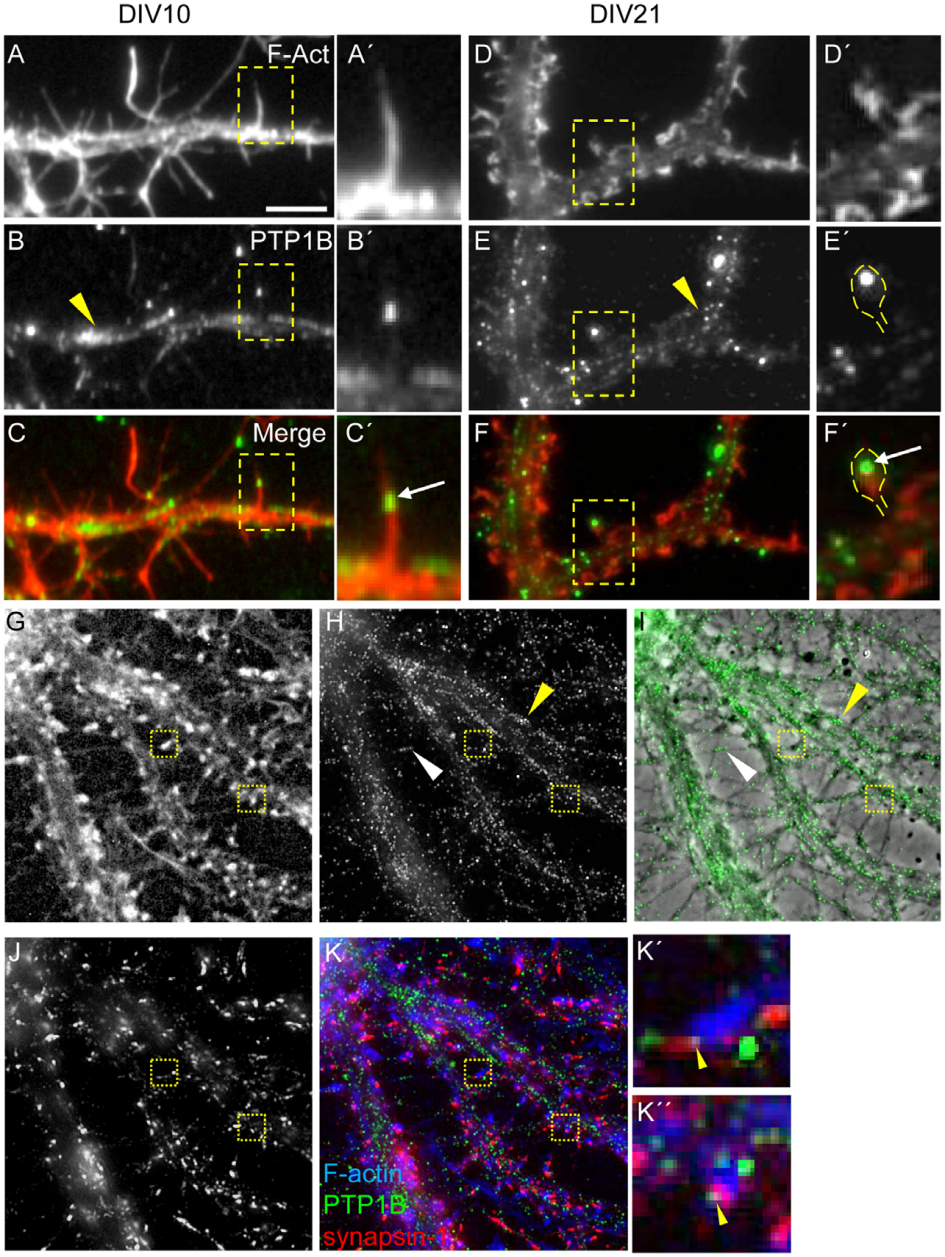
Distribution of endogenous PTP1B during dendrite maturation. Hippocampal neurons from rat embryos were cultured in serum-free medium and fixed at DIV10 (A–C) and DIV21 (D–K). Neurons were processed for fluorescence detection of F-actin, using phalloidin-TMR (A, A’, D, D’) or phalloidin-AMCA (G, K); PTP1B, using a specific mouse monoclonal antibody (B, B’, E, E’, H), and synapsin-1 using a rabbit polyclonal antibody (J). (A–C) At DIV10 most dendritic protrusions display a filopodial shape. (D–F) At DIV21 mushroom-shaped spines prevails. PTP1B displays a punctate distribution in dendritic shafts at both developmental stages (B, E, H, I, yellow arrowheads), and in thin axons (I, white arrowhead). Occasionally, PTP1B puncta locates in dendritic protrusions (white arrows in inset frames C’, F’). In neurons of DIV21, PTP1B puncta sometimes can be seen at the heads of mushroom-like spines (F’), co-localizing with synapsin-1 puncta (boxes, K’, K”, yellow arrowheads). Scale bar, 5 µm.

In situ hybridization of coronal rat brain reveals that PTP1B mRNA is highly expressed in the pyramidal cell layer of hippocampus [27], suggesting a potential role of PTP1B in this plastic brain structure. We recently established that, at the subcellular level, PTP1B protein localizes in dynamic regions of rat hippocampal neurons in culture, such as in filopodia of growth cones, and accumulates in inter-neuronal contacts, suggesting a role in neuronal connectivity [24]. Synaptic connectivity among hippocampal neurons involves interactions between axons and dendrite protrusions called spines [3], [7], [28], [29]. Dendritic spines develop from dynamic filopodia-like protrusions, which are more abundant in initial phases of synaptogenesis (as seen in 10 days *in vitro* (DIV) cultures), while mature spines with morphologically distinct heads and necks (also called “mushroom”) are the hallmark of later stages (e.g. DIV21 cultures). Here we sought to determine whether PTP1B localizes in filopodia-like protrusions and spines; both structures are rich in F-actin and can be easily visualized by phalloidin staining. PTP1B, revealed by antibody staining, is distributed in a punctate pattern in dendritic shafts, filopodia-like protrusions and spines (Figure 1A–F’). PTP1B puncta also showed a scattered distribution along the length of axons which are abundant at this developmental stage (Figure 1H, I). At DIV21, triple staining for PTP1B, synapsin-1 and F-actin, revealed that a small fraction of PTP1B puncta co-localize with synapsin-1 in the head of dendritic spines (Figure 1G–K”). A quantitative analysis reveals that 15.4±1.6% of total filopodia-like protrusions at DIV10, and 16.5±2.3% of total mushroom-like spines at DIV21, contain PTP1B puncta (Table 1). PTP1B was present in 13.0±1.2% of total synapsin-1 puncta, and in 12.0±0.8% of the synapsin-1 puncta colocalizing with mushroom-like spines (Table 1).

**Table 1:** Quantification of the PTP1B presence in dendritic protrusions/spines, presynaptic puncta, and synapses.

*PTP1B in post-synapses*	*% ± SEM*	*N° of neurons*	*N° of protrusions/spines*
DIV10	15.4±1.6	16	976
DIV21	16.5±2.3	25	718
*PTP1B in pre-synapses*			*N° of synapsin clusters*
DIV21	13.0±1.1	10	2037
*PTP1B in synapses*			*N° of synapsin clusters in spines*
DIV21	12.0±0.8	10	608

Dendritic spines were morphologically visualized by F-actin staining with phalloidin and presynaptic puncta were recognized by synapsin-1 staining. Synapses were defined as the overlap of synapsin-1 and spines. The percentage of PTP1B puncta in each condition was calculated.

**Figure 2:**
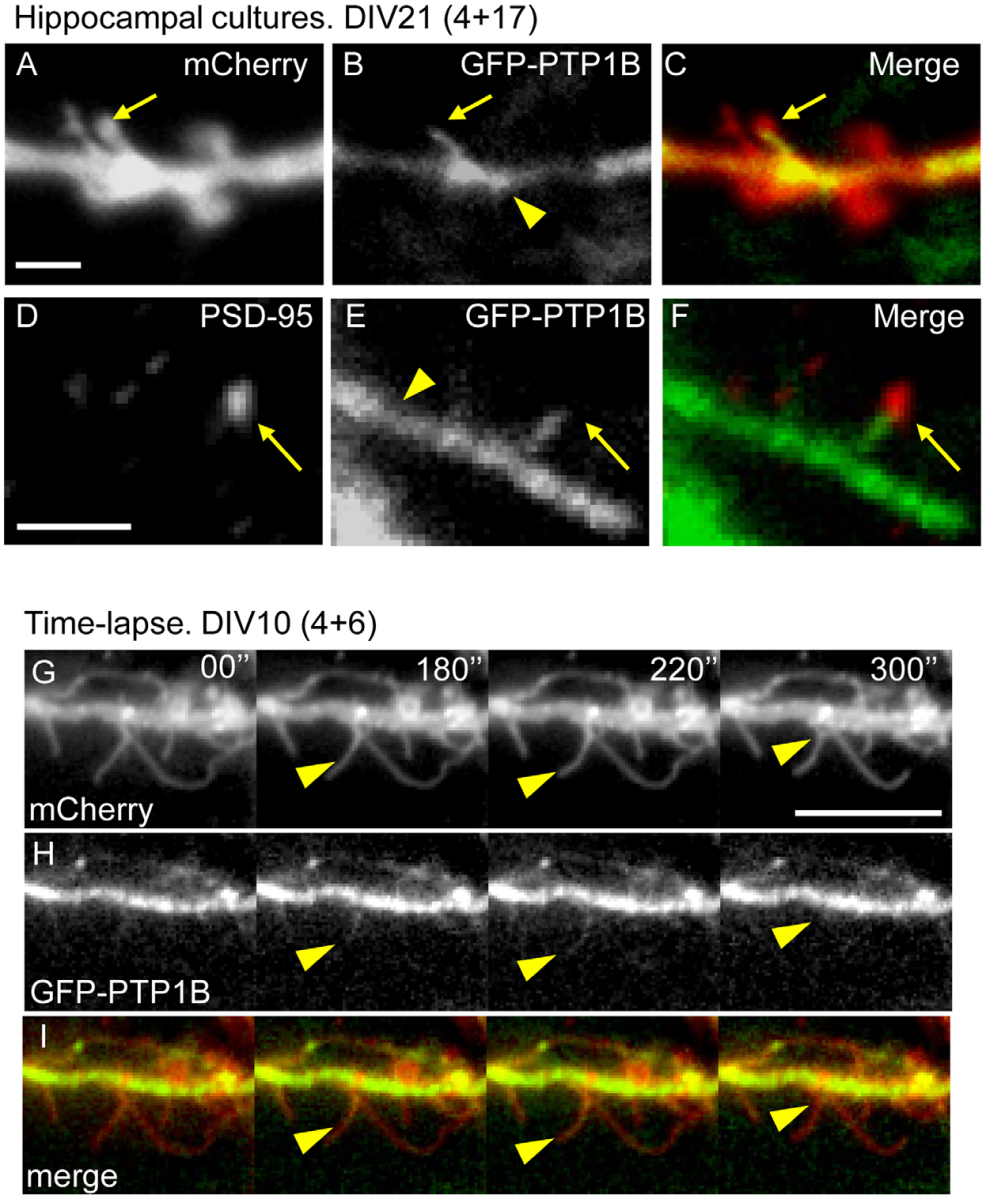
Localization and dynamics of GFP-PTP1B in dendritic protrusions. Hippocampal neurons from rat embryos were co-transfected at DIV4 with plasmids encoding GFP-PTP1B and Lck-mCherry. (B, E, H) GFP-PTP1B signal is relatively strong and uniform in dendritic shafts of neurons imaged at DIV10 (H) and DIV21 (B, E, yellow arrowheads). In the DIV21 cultures, fingerlike protrusions of GFP-PTP1B emerge from dendrite shafts and penetrate into mushroom-like spines detected with the Lck-mCherry (A, B, C, yellow arrows). Scale bar, 2 µm in A. Note that the tips of the GFP-PTP1B protrusions co-localize with PSD-95 clusters detected by immunofluorescence (D, E, F, yellow arrows). Scale bar, 2 µm in D. (G–I) Time lapse studies in DIV10 cultures reveal a dynamic behavior of GFP-PTP1B, entering transiently to preformed dendritic filopodia (yellow arrowheads). Images were taken every 10 seconds during a 10 minute recording. Scale bar, 10 µm in G.

The scarce colocalization with synaptic markers and the low frequency of localization in dendritic spines suggest that PTP1B may be a transient component of this compartment. Likely, PTP1B localization in spines depends on the ER association with microtubules, as demonstrated by several groups including ours [24], [30], [31]. Previous work showed that 1.6% of the total mushroom spines in fixed hippocampal neurons at DIV21 contained microtubules [32]. Observation of living neurons revealed that microtubules transiently invaded dendritic protrusions, including mushroom-like spines, and their presence in spines was detected at a rate of 8.9% per hour [25]. Based on these antecedents we sought to determine whether PTP1B localization in dendritic protrusions was dynamic. Co-expression of GFP-PTP1B and Lck-mCherry in hippocampal neurons of DIV21 revealed, under the channel of the Lck-mCherry fluorescence, the morphology of dendrite shafts throughout and filopodia-like protrusions (Figure 2A). We analyzed neurons expressing the lowest levels of GFP-PTP1B that still can be recorded with our imaging system. A gross estimation of the fluorescence signal after PTP1B immunolabeling, reveals a 3–5-fold increase of the antibody signal in the GFP-PTP1B transfected cells compared to the non-transfected cells (data not shown). GFP-PTP1B distribution was observed as a continuous fluorescence in dendrite shafts and protrusions (Figure 2B, C). This distribution, which differs from the punctate distribution depicted by immunodetection of endogenous PTP1B, is likely related to the higher levels of PTP1B expression in transfected cells [24]. GFP-PTP1B is observed in mushroom-like dendritic spines as fingerlike extensions from dendritic shafts (Figure 2C). Quantitative analysis reveals that GFP-PTP1B extensions are present in 11.4±2.5% of the dendritic protrusions at DIV10 and in 13.5±2.4% at DIV21 (Table 2). These extensions can be found protruding towards PSD-95 clusters detected by immunofluorescence (Figure 2F). Time-lapse analysis revealed that the presence of GFP-PTP1B in the protrusions is transient and frequently disappears within a few minutes (Figure 2, Time-lapse, arrowheads).

**Table 2:** Quantification of the GFP-PTP1B presence in dendritic protrusions/spines.

*GFP-PTP1B in* *post- synapses*	*Filopodia* *(% ± SEM)*	*Thin* *(% ± SEM)*	*Stubby + Mushroom (% ± SEM)*	*Total*	*N° of neurons*	*N° of protrusions/spines*
DIV10	3.9±1.0	6.6±1.9	0.8±0.4	11.4±2.5	15	533
DIV21	3.0±1.0	4.4±1.1	6.0±1.5	13.5±2.4	17	506

Dendritic spines were morphologically visualized by F-actin staining with phalloidin. The percentage of GFP-PTP1B in each condition was calculated.

### PTP1B Regulates Spine Morphology

A well-established role of PTP1B is the stabilization of intercellular unions mediated by N-cadherin [21], [23], [33]. Previous studies have shown that blockade of this type of cell adhesion leads to impaired spine morphology and function [10], [11], [13], [34]–[37]. Taking this into account we reasoned that inhibition of PTP1B activity might have consequences on dendritic spine morphology.

**Figure 3:**
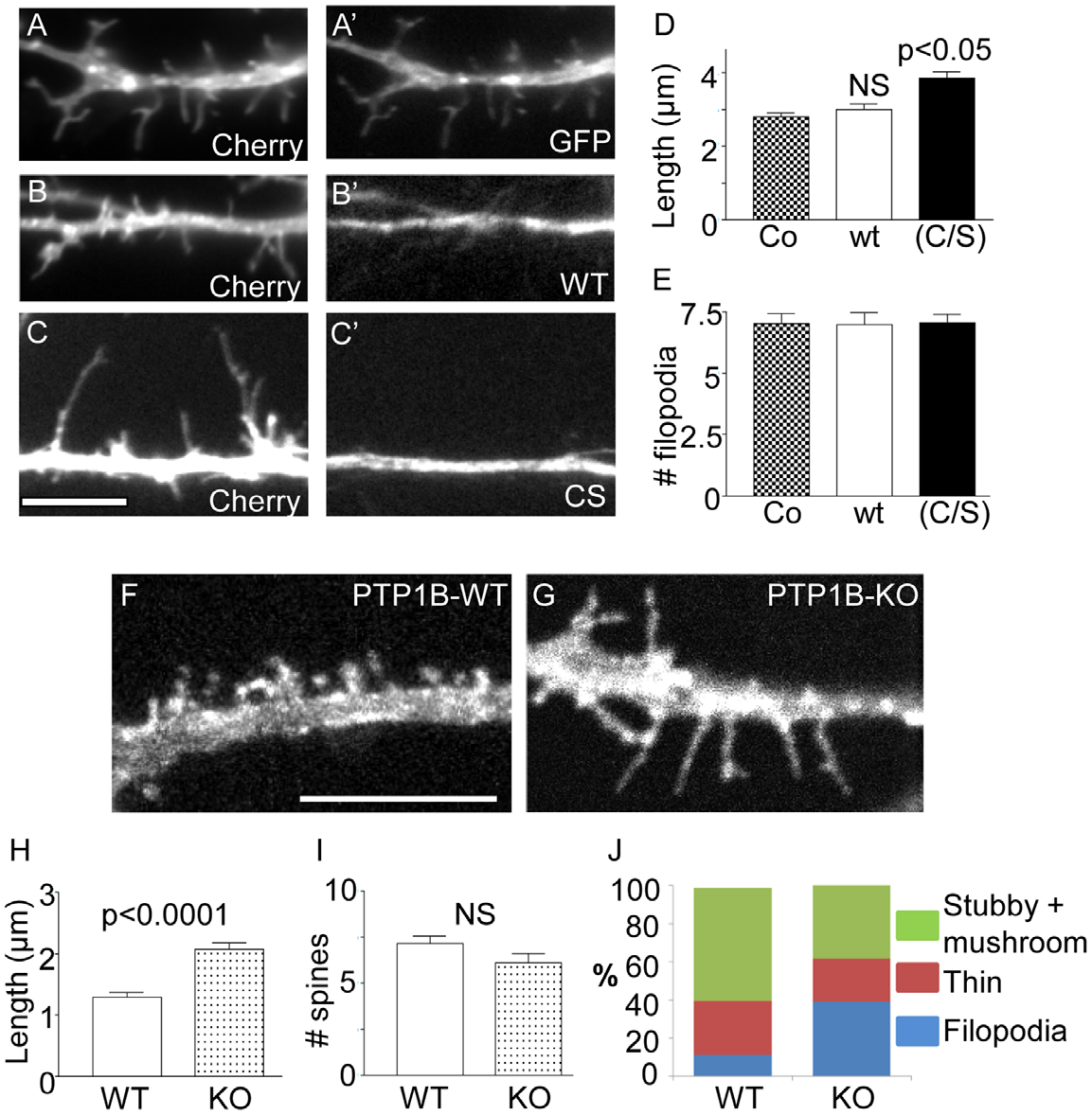
Effect of PTP1B inhibition on dendritic protrusions. (A–E) Hippocampal neurons from rat embryos were co-transfected at DIV 4 with plasmids encoding Lck-mCherry and GFP, GFP-PTP1Bwt or the dominant negative GFP-PTP1B(C/S). At DIV 10 neurons were fixed and imaged for the fluorescent proteins. Expression of GFP-PTP1Bwt (wt) had no effect on filopodia length compared to GFP (B-B’ vs A–A’; scale bar, 5 µm in C). In contrast, expression of GFP-PTP1B(C/S) leads to a significant increase of filopodia length (C–C’). (D) Plot showing the quantification of filopodia length. (E) Plot showing the density of filopodia per 10 µm. (F–J) Hippocampal neurons from PTP1B KO and wild type (WT) newborn mice were transfected at DIV4 with a plasmid encoding Lck-mCherry. At DIV14, neurons were fixed and observed in a fluorescence microscope. Note the predominance of filopodia-like protrusions in the KO neurons (G) compared to WT neurons (F; Scale bar, 5 µm). (H) Quantification of the length of dendritic protrusions shows a significant increase in KO neurons compared to WT neurons. (I) Density of spines does not differ significantly. (J) Quantification of the different morphological types of spines reveals that KO neurons had a significantly reduced proportion of stubby and mushroom spines, and a significantly increased proportion of filopodia-like protrusions, compared to WT neurons. ANOVA p<0.0001 followed by a Dunnett’s post-test p<0.05.

To assess the role of PTP1B in spine morphology, we co-transfected primary rat hippocampal neurons with Lck-mCherry and one of the following constructs: GFP-PTP1B, GFP-PTP1B(C/S) or GFP. In PTP1B(C/S), the essential cysteine 215 at the active site is replaced by serine; this results in a catalytically inactive enzyme which retains the ability to bind substrate similarly to the wild type enzyme, thereby protecting the substrate from dephosphorylation by endogenous PTPs [24], [38], [39], [40]. Neurons were transfected on DIV4 and analyzed 6 days later (DIV10). The overall branching of the dendritic tree was unaffected by the expression of any of these constructs (Figure S1). Quantification of the length of dendritic protrusions revealed an increase of ∼40% in neurons expressing PTP1B(C/S), compared to the GFP expressing control (Figure 3, A, A’, C, C’, D). In contrast, expression of the wild type PTP1B did not significantly affect the length of protrusions (Figure 3, B, B’, and D). The density of dendritic protrusions per 10 µm was not affected by overexpression of WT or PTP1B(C/S) constructs (Figure 3E). These results suggest that PTP1B may be involved in the maturation of dendritic filopodia.

To examine the role of PTP1B in spine morphology further we analyzed primary hippocampal neurons derived from wild type (WT) and PTP1B-deficient (KO) mice [41]. Hippocampal neurons were transfected with Lck-mCherry at DIV4 and fixed for analysis at DIV14 when most neurons show full development of axon and dendrites. Morphological differentiation of axon and dendrites was indistinguishable among WT and KO neurons (Figure S1). Quantification of dendritic protrusion length revealed an increase of ∼60% in KO neurons compared to WT neurons (Figure 3, F–H). These results agree with those obtained in rat neurons expressing the dominant negative PTP1B(C/S). We next examined whether differences in the length of protrusions among WT and KO neurons reflected differences in the proportions of specific morphological types. Dendritic protrusions were categorized following the Spacek & Harris criteria [42]. Briefly, the length and diameter of the protrusions were measured and grouped as “dendritic filopodia” if their length was greater than 2 µm, and “dendritic spines” if their length was less than 2 µm. Additionally, spines were subcategorized as “thin spines” if the length was greater than the diameter, and “mushroom + stubby spines” if their largest diameter was greater than or equal to their length. The latter two groups of spines were considered together because of the difficulty in distinguishing them when they were oriented along the z-axis [42], [43]. We found that mushroom- and stubby-type spines grouped together represented 59.2±2.9% of total spines in WT neurons and 38.9±3.6% in KO neurons (Figure 3J). Thin spines, defined as protrusions with a length/diameter ratio >1, absence of a head at the tip and shorter than 2 µm [42], represented 28.5±2.3% of total spines in WT neurons and 22.3±2.2% in KO neurons. Filopodia-like protrusions, with a length greater than 2 µm, represented 11.1±1.9% of total spines in WT neurons and increased ∼4-fold to 39.3±4.1% in KO neurons (Figure 3J). In agreement with the results obtained expressing the dominant negative PTP1B in rat hippocampal neurons, the density of protrusions in KO hippocampal neurons did not differ with that of WT neurons (Figure 3I). Taken together, these results suggest that PTP1B plays an important role in the morphological maturation of dendritic spines.

### PTP1B Regulates the Organization of Pre-and Post-synaptic Compartments

**Figure 4:**
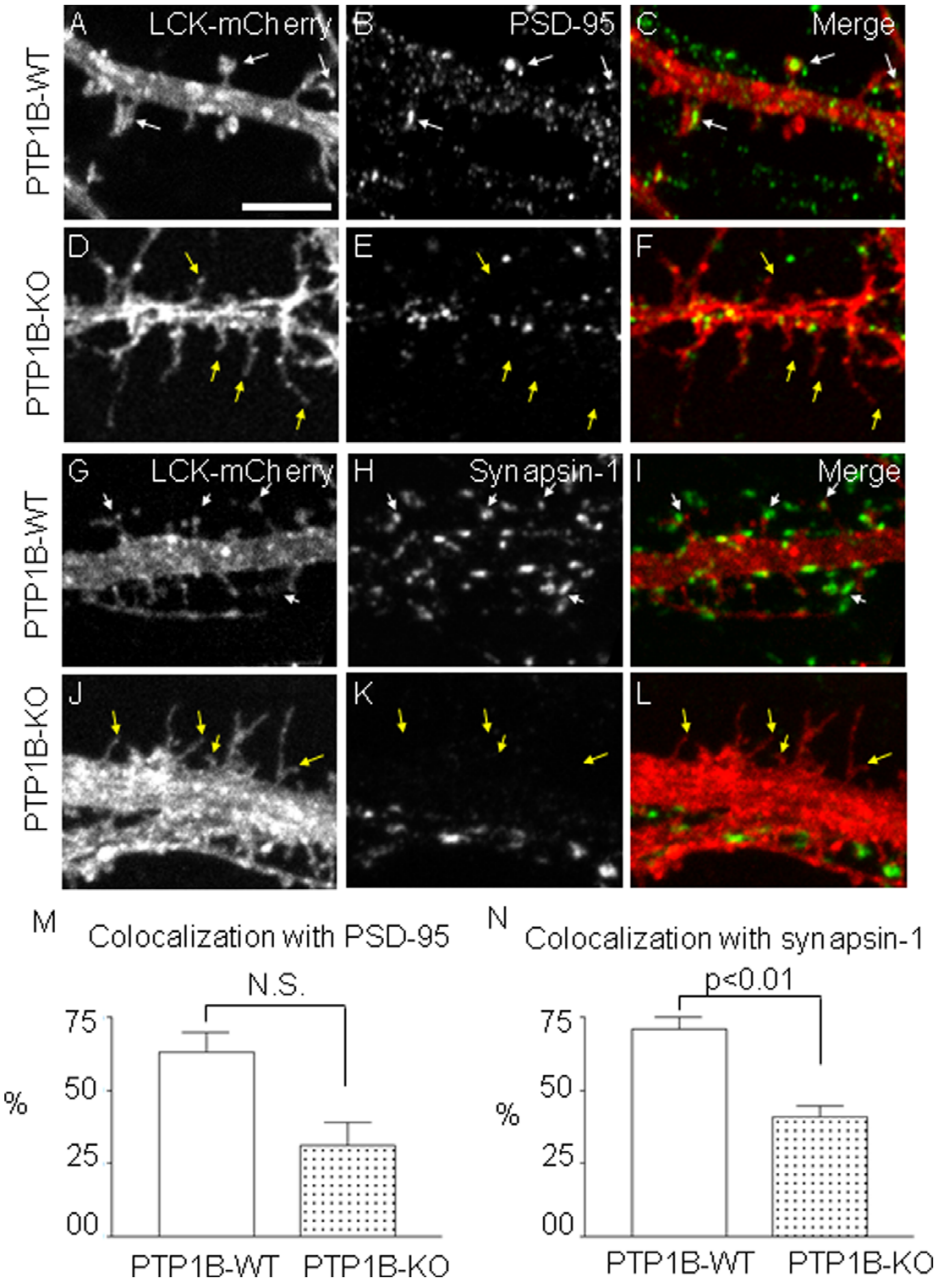
Effect of PTP1B deficiency on the distribution of pre-and post-synaptic markers. Hippocampal neurons from WT (A–C, G–I) and KO (D–F, J–L) newborn mice were transfected at DIV4 with Lck-mCherry to visualize dendritic spines. At DIV14 neurons were fixed and immunostained to detect either the post-synaptic marker PSD-95 (B, E) or the pre-synaptic marker synapsin-1 (H, K). PSD-95 and synapsin-1 were visualized using Alexa Fluor 488-conjugated secondary antibodies. Localization of PSD-95 in spine heads is obvious in WT neurons (A–C, white arrows). In contrast, filopodia-like protrusions from KO neurons show no PSD-95 associated (D–F, yellow arrows). Instead, PSD-95 clusters were found in dendritic shafts. Synapsin-1 clusters are also evident adjacent to spines heads in WT neurons (G–I, white arrows) but not to filopodia-like protrusions in KO neurons (J–L, yellow arrows). Scale bar, 5 µm in A. Plots show the quantification of dendritic protrusions showing colocalization with (M) PSD-95 (WT 63±6.6% versus KO 31±7.7%, p = 0.06) and (N) synapsin-1 puncta (WT 70.8±4.2% versus KO 41.1±3.5%, p = 0.01).

The higher proportion of dendritic protrusions with filopodial morphology in KO neurons predicts alterations in the organization of synaptic elements. To assess synaptic organization, we analyzed the distribution of the post- and pre-synaptic markers PSD-95 and synapsin-1, respectively, in WT and KO neurons expressing Lck-mCherry. PSD-95 is a well established post-synaptic marker that accumulates in the head of mushroom or stubby spines [44]. Dendritic spines with PSD-95 were found in contact with axonal regions containing clusters of synaptic vesicles, which can be visualized by staining synapsin-1, a protein associated with synaptic vesicles [45]. Analysis of DIV14 hippocampal neurons revealed that 63.0±6.6% of total spines in WT neurons, but only 31±7.7% of total spines in KO neurons, contained PSD-95 (Figure 4, A–F, M). Analysis of dendritic protrusions making contact with synapsin-1 clusters were significantly reduced from 70.8±4.2% in WT neurons to 41.1±3.5% in KO neurons (Figure 4, G–L, and N).

### PTP1B Regulates Cadherin Complexes and Beta Catenin Phosphorylation *in vivo*

**Figure 5:**
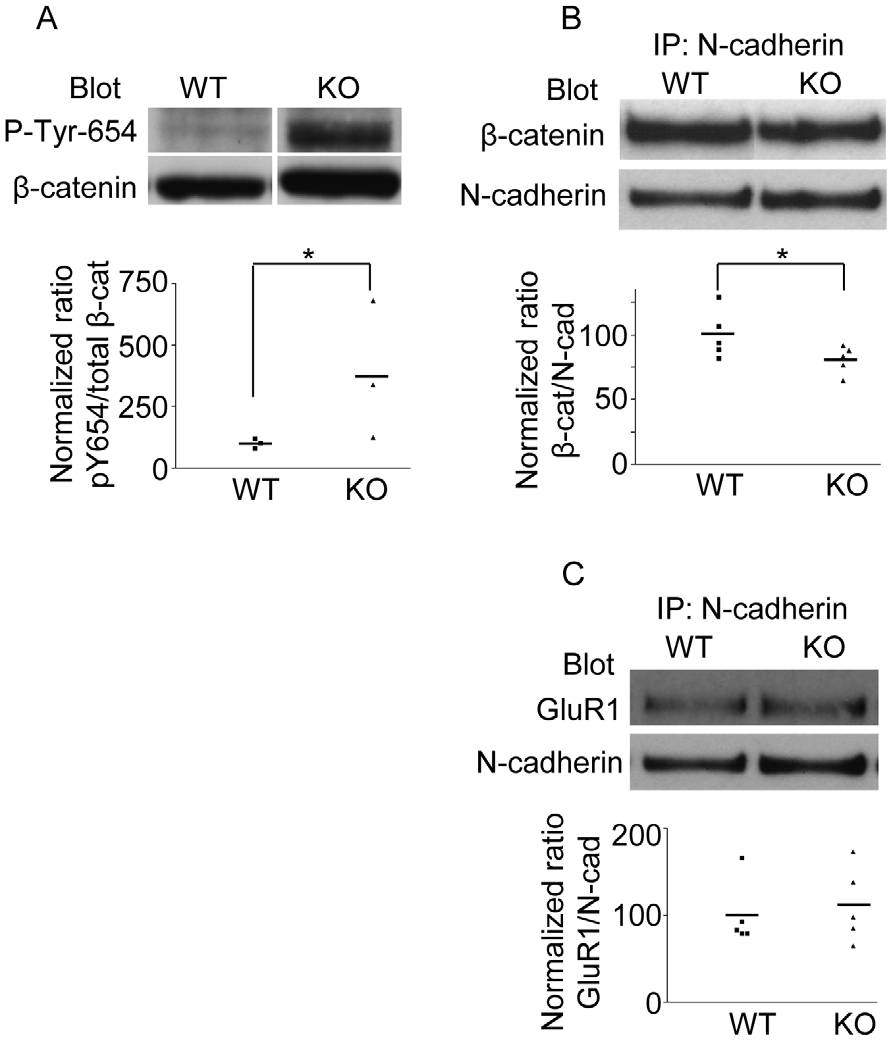
PTP1B controls ß-catenin phosphorylation and association with N-cadherin *in vivo*. Protein extracts from hippocampi of adult WT and KO mice were prepared. (A) Western blots were first probed with a polyclonal antibody specific for ß-catenin-pTyr-654. Subsequently, the membrane was stripped and re-probed with a monoclonal antibody against total ß-catenin. The normalized pY654/ß-catenin signal was calculated from scanned bands. Note that KO mice show a significant increase of the normalized signal compared to the WT mice (KO: 380.5±161.7% vs WT: 100.0±11.6%). (B) N-cadherin was immunoprecipitated using a specific monoclonal antibody. Western blots of N-cadherin immunoprecipitates were first probed to detect total ß-catenin and then re-probed to detect N-cadherin. Experiments from five animals show that normalized ratios of ß-catenin/N-cadherin in KO mice are significantly reduced compared to those in WT mice (KO: 80.8±7% vs WT: 100±3, p<0.05 one-tailed Mann-Whitney test). (C) Amount of GluR1 co-immunoprecipitated with N-cadherin. Experiments from five animals show that normalized ratios of GluR1/N-cadherin are not statistically different between WT and KO mice (KO: 112.1±19.2% vs WT: 100.0±16.8%. Asterisks indicate statistical differences for a p≤0.05, according to the one-tailed Mann-Whitney test.

PTP1B acts to stabilize the association between ß-catenin and N-cadherin in adhesion complexes [21], [23]. Expression of the dominant negative PTP1BC/S in fibroblasts caused the dissociation of ß-catenin from N-cadherin complexes and an increase in ß-catenin phosphotyrosine content [21]. Furthermore, the incubation of cultured retina neurons with permeable peptides that prevent PTP1B binding to N-cadherin produces a similar effect, and is prevented if ß-catenin Tyr-654 is substituted with phenylalanine [23], [46]. These results suggest that PTP1B may be implicated in the dephosphorylation of ß-catenin at Tyr-654. To investigate this possibility *in vivo,* we used a phospho-specific antibody to quantify ß-catenin phospho-Tyr-654 in protein extracts of hippocampus isolated from WT and PTP1B KO mice [41]. Beta-catenin phospho-Tyr-654 was barely detectable in hippocampal lysates from WT mice (Figure 5A). In contrast, ß-catenin from hippocampi of KO animals showed a significant increase in phosphorylation of Tyr-654 (Figure 5A). In contrast, levels of phospho-Erk decreased in KO animals consistent with previous studies (Figure S2) [47]–[49]. It was previously shown that phosphorylation of ß-catenin by pp60^c-src^ significantly decreased the affinity for E-cadherin, decreasing the association constant by 5-fold [17]. Therefore, we predicted that in the absence of PTP1B *in vivo*, ß-catenin would have a reduced ability to form complexes with N-cadherin. We evaluated this possibility by immunoprecipitating N-cadherin from hippocampi of WT and KO mice and immunoblotting for β-catenin. In agreement with our hypothesis, we found a modest (∼30%), but consistent decrease in the amount of ß-catenin in N-cadherin immunoprecipitates from KO mice when compared to WT mice (Figure 5B). These results are the first to show the requirement of PTP1B to maintain the ß-catenin Tyr-654 residue in a non-phosphorylated state *in vivo*, which could be correlated with increased levels of ß-catenin complexed to N-cadherin. For comparison, we also analyzed complexes of N-cadherin with the AMPA receptor subunit glutamate receptor 1 (GluR1), which is not dependent on tyrosine phosphorylation [50]. Experiments from five independent animals do not reveal statistically significant differences in the amount of GluR1 co-immunoprecipitating with N-cadherin between wild type and KO animals (Figure 5C).

### PTP1B is Required for Learning and Memory Consolidation

**Figure 6:**
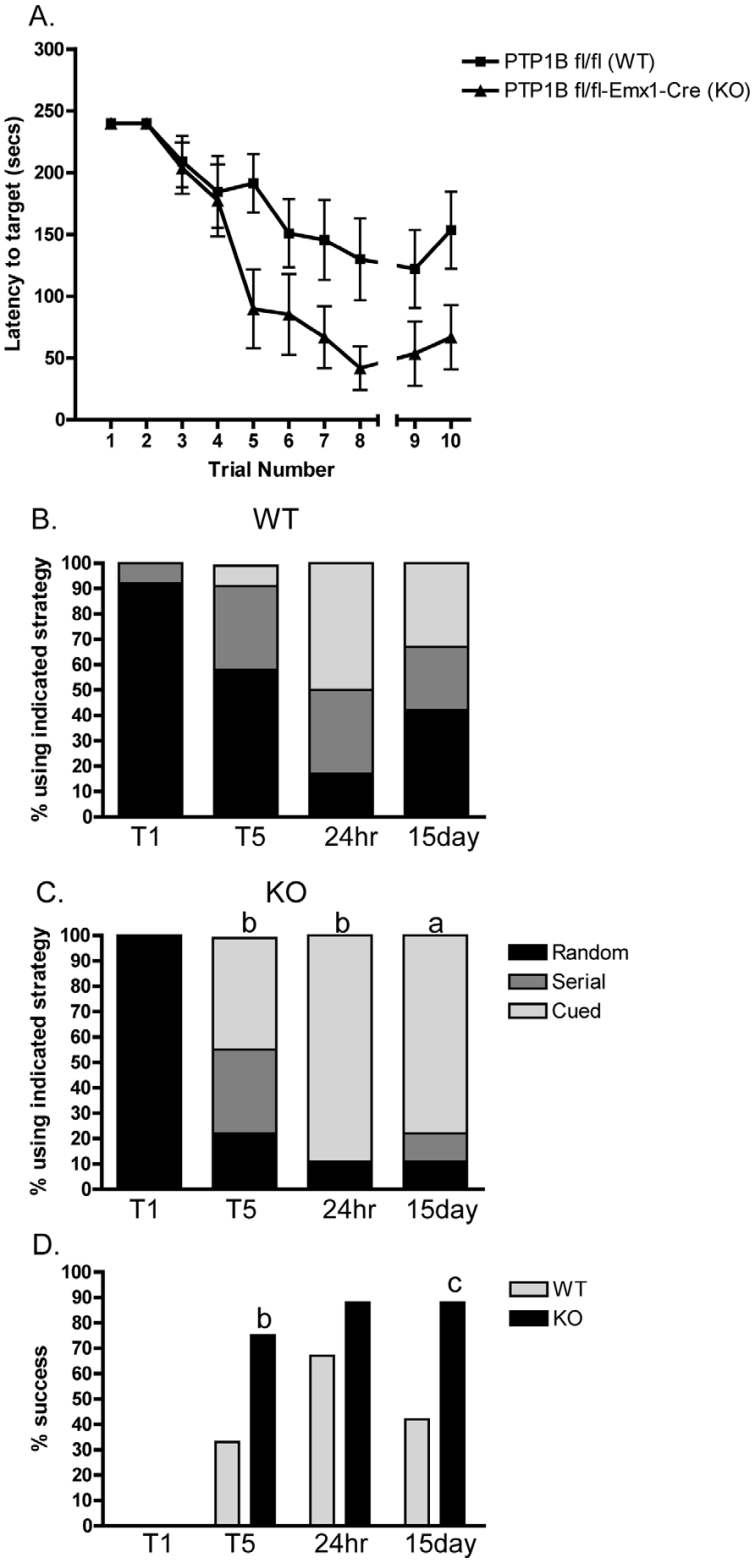
PTP1B fl/fl-Emx1-Cre and littermate PTP1B fl/fl controls were subjected to the Barnes maze. (A) Mice were trained on the maze 2 trials per day for 4 days and their performance plotted as time to enter the target escape hole (trials 1–8). A 24 hour recall (trial 9) and a 15 day recall (trial 10) were performed to assess memory retention. n = 12 WT and n = 8 KO. Data are mean ± SEM, asterisk indicates p<0.01 by 2-way ANOVA. (B and C) Female PTP1B fl/fl (WT; n = 12) and PTP1B fl/fl-Emx1-Cre (KO; n = 8) littermate controls were scored for strategy used in the Barnes maze and plotted as the percentage of mice using a random, serial, or cued strategy to locate the target hole. (D) The percentage of mice successfully locating the target hole in each trial/recall is plotted. T1, trial 1; T5, trial 5; 24 hr, 24 hour recall; 15 day, 15 day recall. a, p<0.05; b, p<0.08; c, p = 0.06 by a nominal logistic followed by a Pearson’s Chi-square test comparing WT and KO for the indicated trial.

Alteration of the phosphorylation balance in the synapse can affect synaptic plasticity [51]. However, its impact on learning and memory has been more difficult to evaluate. In mice lacking the tyrosine kinase Fyn, or the receptor protein tyrosine phosphatase RPTPα, the induction of LTP and spatial learning capabilities are impaired, but these mice also show alterations in the development and morphology of the hippocampus [52], [53]. In order to assess whether PTP1B-deficiency alters learning and memory, mice lacking PTP1B specifically in the hippocampus and cortex (PTP1B^fl/fl^-Emx1-Cre) were generated (Figure S3). In the Barnes maze, a paradigm requiring mice to learn, female PTP1B^fl/fl^-Emx1-Cre mice had improved performance compared to WT (PTP1B^fl/fl^) littermate controls (Figure 6A). Similar results were seen in male mice (data not shown). Furthermore, PTP1B^fl/fl^-Emx1-Cre mice consistently utilized a more efficient strategy (cued) and had a better overall success rate and 15 day recall compared to WT controls (Figure 6, B–D). These results demonstrate a novel role for forebrain PTP1B in learning and memory, and are consistent with the elevated phospho-Tyr-654 ß-catenin levels noted in hippocampal lysates.

## Discussion

### PTP1B Localization and Contribution to Dendritic Spine Differentiation

PTP1B mRNA is highly expressed in the hippocampus [27]. In addition, studies in cultured hippocampal neurons showed that impairment of PTP1B function negatively affects axon and dendrite growth, as well as growth cone dynamics [24], [54]. These results suggest that PTP1B may play a positive role in the development of productive synaptic contacts in the hippocampus. Indeed, in this paper we show that PTP1B is present in dendritic spines and co-localizes with the pre-synaptic marker synapsin-1. Our studies using GFP-PTP1B suggest a transient localization of PTP1B in dendritic spines, as it was recently observed for dynamic microtubules [32], [55]. We and others have shown a tight association between the ER and microtubules in hippocampal neurons [24], [30], [31]. The possibility that microtubules could regulate the localization of ER-bound PTP1B in dendritic protrusions remains to be determined. Also, whether the number of spines invaded by PTP1B is enhanced by neuronal activity, as it was found for microtubules [25], remains an interesting possibility to be addressed.

Here we found that impairing the expression of PTP1B in hippocampal neurons, by dominant negative or by gene targeting approaches, increased the proportion of filopodia-like protrusions, a hallmark of an immature stage of synaptogenesis, without affecting the density of total protrusions. Since dendritic protrusions are continuously appearing and disappearing [29], [56], [57], this result suggests that PTP1B is required for the normal maturation of dendritic spines and likely does not inhibit the turnover and genesis of protrusions. Furthermore, this result may at least partly explain the reduction of synapsyn-1 and PSD-95 localization in dendritic protrusions of neurons lacking the expression of PTP1B. The molecular target(s) of PTP1B involved in the morphological differentiation of spines are presently unclear although the cadherin adaptor ß-catenin is a likely candidate [16], [58].

### Role of PTP1B in Regulating β-catenin Function

It is well-established that cadherin complexes contribute to spine morphogenesis, plasticity and function [6]–[8], [59]. Beta-catenin, a major intracellular binding partner of cadherin, associates with the cytoplasmic domain of cadherin and with α-catenin, mediating interactions with the actin cytoskeleton [60]. Post-synaptic loss of ß-catenin in hippocampal neurons using the cre/loxP technology results in profound alterations in spine morphology, leading to elongated spines and a reduced proportion of mushroom spines, with no alterations in spine density [61]. These morphological alterations are remarkably similar to the phenotype of PTP1B KO neurons described in the present work (Figure 3). Another study found that suppression of ß-catenin expression in hippocampal neurons did not have an effect on the size of PSD-95-GFP puncta [62]. In PTP1B KO neurons we also did not find an obvious change in the size of PSD-95 clusters; rather, we observe a redistribution of these clusters to the dendritic shafts (Figure 4).

Beta-catenin localization and function in dendritic spines is dynamically regulated by its phosphorylation state [35], [63]. Membrane depolarization induces a redistribution of ß-catenin from dendritic shafts into spines, increasing its association with cadherin and likely enhancing cadherin-mediated adhesion. This process is enhanced in the presence of a non-phosphorylatable Y654F-ß-catenin mutant, and is inhibited in the presence of a phosphorylation-mimic Y654E mutant [35]. Thus, the non-phosphorylated form of ß-catenin at the critical residue Tyr-654 seems to be required for proper organization of the post-synaptic density and function.

PTP1B dephosphorylates ß-catenin in N-cadherin complexes [16], [33], [64]. Inhibition of PTP1B in different cell types leads to loss of N-cadherin-mediated adhesion, dissociation of ß-catenin from N-cadherin and accumulation of phosphorylated ß-catenin in the cytosol [21], [23]. Since the affinity of ß-catenin for cadherin is reduced by phosphorylation of Tyr-654 [17], a finding that is supported by structural analysis of cadherin/ß-catenin co-crystals [65], it is likely that PTP1B targets this residue. In fact, in this paper we show for the first time that PTP1B is required for ß-catenin-Tyr-654 dephosphorylation *in vivo*. As expected for a direct enzyme/substrate relationship, ß-catenin phospho-Tyr-654 is significantly elevated in hippocampi of PTP1B−/− mice. Importantly, this result correlates with a modest but consistent reduction of ß-catenin association with N-cadherin, an event that is crucial for cadherin function. Thus, lack of PTP1B would, to some extent, mimic the depletion of ß-catenin expression, which in concordance with our results, also results in an alteration of spine morphology [61].

### Role of PTP1B in Learning and Memory

The results of the present work add to a growing body of empirical evidence highlighting a correlation between the structure and number of spines with the processes of learning and memory; however, the underlying mechanisms remain enigmatic. A network of regulatory proteins may impinge in the actin cytoskeleton and cell adhesion complexes, underlying structural and morphological changes that may affect learning and memory [1], [66]. Since a lack of PTP1B perturbs normal N-cadherin function, it is conceivable that there is also an effect on filopodia maturation to spines. Our hypothesis is that PTP1B modulates this process but is not essential. Thus, neurons from PTP1B KO mice may display morphological features characteristic of an “immature brain”, which are also more prone to learn. Our results are consistent with a recent study which found that mice with conditional ablation of the *Dicer1* gene in the adult forebrain outperform wild type animals in a variety of learning and memory tests [67]. Lack of the *Dicer1* gene increases post-tetanic potentiation and the length of dendritic spines in CA1 hippocampal neurons. The increase of long filopodia-like spines in dendrites of the mutant neurons suggests an active remodeling of synapses that could facilitate the improvement of memory. Another recent study showed that mice deficient in expression of the receptor protein tyrosine phosphatase sigma exhibit enhanced novel object recognition memory and this correlates with increase in the length of dendritic protrusions [68].

Along with cytoskeletal changes in dendritic spines, synaptic plasticity during learning and memory also results in strengthening and weakening of pre- and post-synaptic contacts, a process driven by cell adhesion molecules such as cadherins. Beta-catenin is highly expressed in the adult mouse amygdala and is dynamically regulated at both the transcriptional and post-translational levels with fear learning [19]. Pharmacological stabilization of ß-catenin with lithium chloride resulted in enhanced learning, while genetic deletion of the gene that encodes ß-catenin in the amygdala resulted in impaired learning. In both cases, the manipulation affected the consolidation, but not acquisition, of the fear memory [19], [69]. Interestingly, the phosphorylation of ß-catenin-Tyr-654 in amygdala significantly increased during the first 30 min after fear training, and then follows a phase of decrease until 2 h where it starts rising again [19]. Thus, the affinity of ß-catenin for cadherin in the amygdala seems to be dynamically regulated during fear consolidation. These changes in ß-catenin-Tyr-654 phosphorylation inversely correlate with the amount of ß-catenin complexed with cadherin.

**Figure 7:**
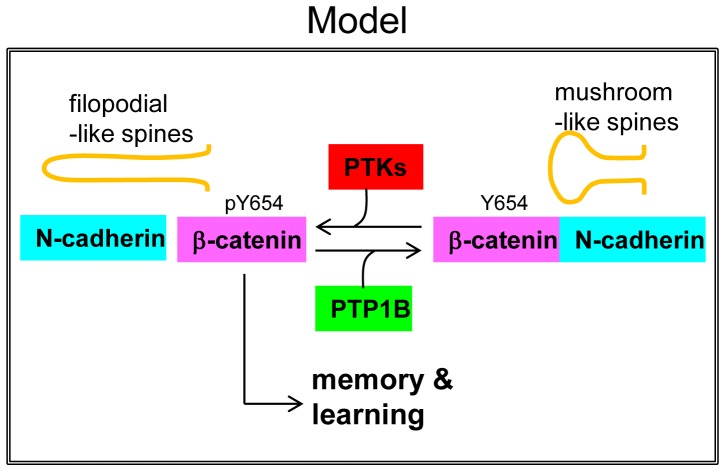
Model representing the potential function of PTP1B in the hippocampus. PTP1B dephosphorylates ß-catenin at the residue Tyr-654, opposing the activity of protein tyrosine kinases. This function of PTP1B ensures ß-catenin association with N-cadherin in functional adhesion complexes, and is required for normal differentiation of mushroom-like spines. Loss of PTP1B expression/function would favor a stage characterized by morphologically immature (and likely more dynamic) spines, which may positively modulate memory and learning processes.

We found that mice with PTP1B deletion in the hippocampus and cortex (PTP1B^fl/fl^-Emx1-Cre) displayed significantly improved performance compared to WT mice in the Barnes maze (a paradigm requiring mice to learn). Furthermore, PTP1B^fl/fl^-Emx1-Cre mice utilized a more efficient strategy (cued) and had a better overall success rate and recall compared to WT controls. Although we did not determine the phosphorylation of ß-catenin-Tyr-654 in the trained animals, we found that the phosphorylation of this residue was already significantly increased in control PTP1B^fl/fl^-Emx1-Cre mice. Accordingly, we also detected a reduction of ß-catenin/N-cadherin complexes in the hippocampus (Figure 5). These results combined with the filopodia-like phenotype of spines in PTP1B KO animals, suggest that lack of PTP1B promotes molecular and cellular conditions that may prime animals for enhanced learning. A possible scenario including our present observations is shown in a model (Figure 7). Importantly, the finding that mice with forebrain-specific PTP1B-deficiency show improved spatial learning and enhanced memory retention demonstrates a novel role for this phosphatase.

Other protein tyrosine phosphatases have also been implicated in learning and memory. The striatal-enriched protein tyrosine phosphatase (STEP) KO mice show improved hippocampal learning and memory; STEP is expressed in the hippocampus, cortex and striatum, and the isoform STEP61, localizes in the ER [70]–[72]. Furthermore, recent work showed that mice deficient in expression of the receptor protein tyrosine phosphatase sigma exhibit enhanced novel object recognition memory [68]. Thus, it is likely that the combined activity of several tyrosine kinases/phosphatases modulate learning and memory in a dynamic fashion. An exciting challenge will be to identify novel targets of these kinases and phosphatases and understand how their spatiotemporal regulation can be causally related to these highly integrated and complex brain processes.

## Materials and Methods

### Ethics Statement

All animal care protocols and procedures were approved by the University of Pennsylvania Institutional Care and Use Committee.

### Materials

DMEM, Neurobasal™ Medium, N2, B27, L-glutamine, penicillin-streptomycin, trypsin, bovine fetal serum and horse serum were from Invitrogen (Carlsbad, CA). Poly-L-lysine and ovoalbumin were from Sigma-Aldrich (St. Louis, MO). Coverslips were from Marienfeld GmbH & Co. (Lauda-Königshofen, Germany). Fibrilar actin was detected using either phalloidin-TRITC (1/1000), or phalloidin-AMCA (1/200), both from Invitrogen.

### Antibodies

The following antibodies were used for immunofluorescence: mouse monoclonal antibody against PTP1B (1/200) from Calbiochem (EMD Biosciences, San Diego, CA); mouse monoclonal antibody against PSD-95, clone 6G6-1C9 (1/500) form Thermo Scientific Pierce Antibodies (Rockford, IL); rabbit polyclonal antibody against synapsin-1 (1/1000) from Sigma-Aldrich (St. Louis, MO). Primary antibodies in the epifluorescence studies were detected using Alexa Fluor 488- and Alexa Fluor 546-goat conjugated secondary antibodies (1/500) from Invitrogen. In the preparations for confocal microscopy we used DyLight 488-goat conjugated secondary antibodies from Jackson Immunoresearch (West Grove, PA). For Western blots and immunoprecipitation studies we used the following antibodies: rabbit polyclonal antibody against the phosphorylated tyrosine 654 of ß-catenin (1/100) from AbCam (Cambridge, MA); mouse monoclonal antibody against ß-catenin, clone 14 (1/2000) from BD Biosciences (Franklin Lakes, NJ); mouse monoclonal antibody against N-cadherin, clone GC-4 (1/2500) from Sigma-Aldrich, rabbit polyclonal antibody against glutamate receptor1, clone GluR1 (1/2500) from Millipore (Billerica, MA), rabbit anti-phospho-p44/42 MAPK, clone #9101, rabbit anti-p44/42 MAPK, clone #4695, both from Cell Signaling Technology (Danvers, MA). Secondary antibodies used in blots (1/5000) were the HRP-conjugated donkey anti-rabbit IgG and HRP-conjugated sheep anti-mouse IgG from GE Healthcare Life Sciences (Pittsburgh, PA).

### Cell Culture Procedures

Dissociated hippocampal primary cultures were prepared from rat embryos or newborn mice as described in Goslin and Banker, 1991 [73], with modifications (see below). Female pregnant Sprague-Dawley rats were obtained from the Veterinary Faculty of the University of Buenos Aires and the brains from 18-day-old rat fetuses were used. PTP1B KO mice were generated previously [41]. To obtain newborn pups from WT and PTP1B-KO breeders, animals of 3 to 6 months old were mated. Animals were genotyped before the crossings as described [74]. To obtain single cell cultures, tissue was treated with 0.25% trypsin in (HBSGK; 20 mM HEPES, 150 mM NaCl, 2 mM glucose, 3 mM KCl, pH 7.4) for 15 min at 37°C. A single-cell suspension was prepared in Neurobasal™ Medium containing 0.3 mg/ml glutamine, 100 units/ml penicillin, 100 µg/ml streptomycin and 10% (v/v) horse serum. Cells were plated at desired concentrations (ranging from 5,000 to 50,000 cells per cm^2^) on coverslips coated with 0.8 mg/ml poly-L-lysine prepared in borate buffer 0.1 M pH 8.5. After 2 h in a 5% CO2 humidified incubator (37°C), medium was changed to serum free Neurobasal™ Medium supplemented with 0.5 mg/ml ovalbumin, N2 and B27. Cells were maintained in the incubator for 1 to 21 days before fixation and imaging.

### DNA Constructs and Transfection Procedures

Lck-mCherry was kindly provided by Steve Green (University of Iowa). GFP-PTP1B and GFP-PTP1B-CS plasmids were described previously [38]. Hippocampal neurons were transfected in 35 mm dishes at DIV4 using the calcium phosphate method [75]. Briefly, the conditioned neuronal culture medium was aspirated and saved for subsequent use. The transfection medium (Neurobasal with 15 mM HEPES, 0.3 mg/ml L-glutamine, 10 mM glucose, pH 7.5) was added and cells were incubated for 30 minutes before adding the CaCl2/plasmid complexes. These complexes were prepared by mixing 3 µg DNA in 18.6 µl of a solution of 250 mM of CaCl_2_. This solution was dropped to an equal volume of transfection buffer (50 mM BES, 1.5 mM Na_2_HPO_4_, 280 mM NaCl, pH 7.1), then mixed by bubbling 5 times with the pipette tip, and immediately added to the neurons. After 3 hours of incubation, the transfection medium with the precipitates was removed and cells were washed 3 times with HBSGK. The saved conditioned neuronal culture medium was added back to the dishes and cells were cultured until use. In the case of double transfections the plasmids were mixed before the addition of CaCl_2_.

### Immunofluorescence

Dispersed single cells were fixed at indicated times with 4% paraformaldehyde and 4% sucrose in PBS for 20 min, permeabilized with 0.5% Triton X-100 in PBS for 10 min at room temperature, and blocked with 3% BSA in PBS overnight at 4°C. Incubations with the primary and secondary antibodies were carried out in a humid chamber for 1 h at 37°C. Samples were mounted in PBS/glycerol (1∶1, vol/vol) and observed through a 100×/1.4 NA objective in a Nikon E600 microscope (Melville, NY) coupled to a Spot RT Slider CCD camera (Diagnostic Instruments, Sterling Heights, MI). Red and green fluorescence was detected using Nikon B-2E/C and G-2E/C filter sets, respectively. For confocal imaging we used a Leica SP5 spectral imaging confocal/multiphoton microscope with a HCX PLAPO 63×/1.4 NA objective. For scanning of dendritic sections we did z-stacks and the pinhole was set up to 1 Airy disk to get a step size of 300 nm. Regions were zoomed to a pixel size of 63 nm. Sequential line scanning was taken at a 100 Hz speed using 488 nm Argon and 543 nm HeNe lasers for excitation, detecting the signal with a Hamamatsu R6357 PMT. For quantification, maximum projection of the z-stacks was used. Wide-field and confocal images were analyzed with the ImageJ software (Wayne Rasband, NIH, Bethesda, MD, USA).

### Image Analysis and Quantification

Quantifications were performed in segments of 20 µm length defined from primary dendrite branches of neurons cultured for DIV14 and DIV21. To quantify the proportion of dendritic protrusions containing endogenous PTP1B, regions of interest (ROIs) including the whole protrusion as detected by phalloidin staining, were copied and pasted into the PTP1B stained images. ROIs containing at least one fluorescent puncta of PTP1B were counted as positives. Only PTP1B puncta with a brightness level of at least twice the background signal in non cellular areas were considered. For quantification of synapsin-1 clusters containing PTP1B we created ROIs around the clusters and pasted them into the PTP1B stained image, the same ROIs were also pasted in the phalloidin image for determining the synapsin-1 clusters associated with dendritic spines. Quantification of co-localization of the protrusions with synaptic markers was performed in neurons transfected with Lck-mCherry and processed for immunofluorescence of synapsin-1 or PSD-95. ROIs encircling protrusions were obtained as described before and pasted on either PSD-95 or synapsin-1 stained images. The presence of clusters inside the mask was counted as a positive event.

To quantify the length and density of dendritic protrusions we used neurons transfected with Lck-mCherry. To measure length, a linescan was traced from the base to the distal tip of the protrusion and the length value was obtained using ImageJ. Results were expressed per 20 µm of dendritic segment. The effect of expressing GFP, GFP-PTP1B-WT and GFP-PTP1B(C/S) in dendritic spines was analyzed in rat hippocampal neurons displaying similar levels of fluorescence of the GFP proteins, judged by the intensity levels of images obtained with the same exposure and acquisition conditions.

### Dendritic Spine Categorization

Dendritic protrusions from DIV14 hippocampal cultures obtained from WT and KO mice were categorized following the Spacek & Harris criteria [42]. Briefly, the length and diameter of the protrusions were measured and grouped as “dendritic filopodia” if their length was greater than 2 µm, and “dendritic spines” if their length was less than 2 µm. Additionally, spines were subcategorized in “thin spines” if the length was greater than the diameter, and “mushroom + stubby spines” if their diameters were greater than or equal to their lengths. The latter two groups of spines were considered together because of the difficulty to distinguish among them when they appeared oriented in the z-axis of the z-stack [42], [43].

### Time-lapse Imaging

Cells in glass-bottom dishes were placed on the stage of a Nikon TE2000 inverted microscope enclosed within a microscope incubator system (Solent Scientific Ltd, Fareham, UK) that maintained the temperature at 37°C during the whole experiment. Imaging medium was phenol red-free DMEM with high-glucose, supplemented with 4 mM L-glutamine and 25 mM Hepes buffer, 10% fetal bovine serum, and antibiotics. The medium also contained 0.5 U/ml oxyfluor (Oxyrase, Inc., Mansfield, OH) to prevent photobleaching and photodamage. Hippocampal neurons were imaged with a 60×/1.4 NA Plan Apo objective. The excitation light was attenuated using ND8 neutral density filters. Images were captured with an Orca-AG cooled CCD camera (Hamamatsu Photonics, Hamamatsu, Japan) using 2×2 binning with exposure times of 200 milliseconds. EGFP and Lck-mCherry were detected using filters (Chroma Technology Corp, Brattleboro, VT) placed in filter wheels; EGFP (excitation 470/20, emission 525/40) and Lck-mCherry (excitation 565/25, emission 620/60), using a 86007bs multi-band dichroic mirror. Illumination was shuttered using SmartShutters coupled to a Lambda 10-B controller (Sutter Instrument, Novato, CA). Under our experimental conditions, we did not detect significant photobleaching. All peripherals were controlled with Metamorph software (Molecular Devices, Downingtown, PA). For analysis of the GFP-PTP1B dynamics in dendrites, consecutive images of the red and green channels were acquired every 10 seconds for at least 10 minutes, and stacks were built using the Metamorph software.

### Western Blot and Immunoprecipitation

Mouse hippocampi were homogenized on ice in RIPA buffer (10 mM Tris.HCl, pH 7.4; 150 mM NaCl; 0,1% SDS; 1% Triton X-100; 1% deoxycholate; 5 mM EDTA), supplemented with a cocktail of protease inhibitors and phosphatase inhibitors (2 mM Na-orthovanadate, 10 mM sodium pyrophosphate, 10 mM ß-glycerophosphate, 50 mM NaF), from Sigma-Aldrich. Homogenates were incubated at 4°C for 30 minutes with rotation and centrifuged at 12000×g for 10 minutes. Protein concentration of the supernatants was measured with the Thermo Scientific Pierce BCA protein assay. Sample buffer with ß-mercaptoethanol (Fisher Scientific) was added to the lysates, boiled for 10 minutes and proteins were separated by SDS-PAGE and transferred to polyvinyl difluoride (PVDF) membranes. Membranes were blocked for 1 hour with 5% BSA in TBS (50 mM Tris.HCl pH 7.8; 150 mM NaCl) and incubated over night at 4°C with primary antibodies diluted in blocking solution. After three washes with TBST (TBS with 0.1% Tween-20) membranes were incubated with HRP-conjugated secondary antibodies and revealed by ECL. For stripping, blots were incubated (30 min, 50°C) with TBS containing 100 mM ß-mercaptoethanol and 2% SDS, blocked and re-probed. For immunoprecipitations, the homogenates were prepared in mild lysis buffer (MLB: 20 mM Tris.HCl pH 7.4; 150 mM NaCl) containing 1% NP40 and a cocktail of proteases and phosphatases inhibitors (Sigma-Aldrich) [76]. Lysates were centrifuged and 0.8 mg of protein from the supernatant was incubated overnight at 4°C with 1 µg of anti- ß-catenin antibody. Then, protein A conjugated to agarose beads was added and incubated for 2 additional hours. Agarose beads were washed twice with MLB followed by a final wash in buffer without detergent. All incubations were conducted at 4°C. Agarose beads were mixed with sample buffer and analyzed as described above. Semi-quantitative analysis of the intensity of the bands was performed using ImageJ software. Briefly, a ROI encircling each band was created, and the average intensity of the mask minus background was multiplied by the area. Values of phospho-Tyr-654 β-catenin signals were divided by values of total ß-catenin obtained after membrane stripping. For N-cadherin immunoprecipitations, values for ß-catenin and GluR1 signals were divided by values of total N-cadherin signal obtained after membrane stripping. Data were expressed as percentage of every individual band (WT or KO ratios) referred to the average of WT ratios in the same membranes used.

### Animal Care

All animal care protocols and procedures were approved by the University of Pennsylvania Institutional Care and Use Committee. We maintained mice on a 12-h light-dark cycle in a temperature-controlled barrier facility, with free access to water and food (standard chow autoclavable Lab Diet #5010). Age-matched littermates were used for all experiments. All mice are on a mixed Sv129/C57Bl6 background. PTP1B−/− mice were generated by crossing PTP1B fl/fl mice to a “deleter”-Cre (CMV-Cre; JAX stock #006054) to ultimately obtain mice with germline PTP1B-deficiency. PTP1B+/+ and PTP1B−/− mice were generated by crossing PTP1B+/− breeders. Mice with forebrain-specific deletion were generated by crossing PTP1B fl/fl mice [77] with Emx1-Cre mice (JAX stock #005628 [78]) to generate PTP1B +/fl-Emx1-Cre breeders. PTP1B +/fl-Emx1-Cre mice were then crossed with PTP1B fl/fl mice to generate PTP1B fl/fl (WT) and PTP1B fl/fl-Emx1-Cre (KO) mice for experiments. Mice were genotyped for the floxed *Ptpn1* allele and Cre as described [74].

### Modified Barnes Circular Maze

The Barnes maze is a useful test to examine learning behaviors and strategies [79], [80]. The maze consisted of a black circular disc (90 cm in diameter) with 24 holes (5 cm in diameter) around the perimeter and an escape box (15 cm×8 cm×7 cm) located under one of the holes. The Barnes maze used in these experiments has been described in detail elsewhere [80]. The disc was elevated 70 cm above the floor and situated in a room with white walls. Visual cues were located on each of 3 separate walls consisting of a black and white checkerboard, two red circles, and blue and white stripes. All cues were (55.8×71.1 cm). Each cue was positioned 80 cm above the floor and 15 cm from perimeter of maze. WT (PTP1B fl/fl) and KO (PTP1B fl/fl-Emx-Cre) naïve mice at 5 months of age were used for this study. Mice were trained in 2 trials per day for 4 days (Trials 1–8). Trials within each day were separated by 4 h. A video camera was used to record the behavior of mice during all trials and tests. The time to enter the escape box was scored in each trial. To address the complex intersection between learning and motivation that occurs in any repeated task, a mild novel stimuli was introduced daily during training (a bright light (400 l×), a fan on the medium setting, or a novel noise (100 dB) were positioned 35 cm above center of maze). For each trial, the mouse was placed under a glass beaker in the center of the maze for 15 seconds prior to trial start. The mouse was allowed 4 minutes to find the escape box in each trial. The mouse was allowed to remain in the escape box for 30 s prior to transfer back to the home cage. The entire apparatus was thoroughly cleaned with water and dried between each mouse. As previously described, search strategies were classified as random, serial, or cued [80], [81]. To examine genotype differences on memory performance, a recall trial was performed 24 hours (trial 9) and 15 days (trial 10) following the last exposure to the maze. Conditions were identical to those of acquisition test exposure.

### Data Analysis

Data are presented as mean ± SEM. Length and density of protrusions of neurons transfected with GFP, GFP-PTP1B-WT and GFP-PTP1B(C/S) were compared using 1-way ANOVA. Statistically significant differences between groups (p<0.05) were further analyzed by a Dunnett’s post hoc test. Data relating to length and density of neurons isolated from WT and KO mice were compared using a two tailed Student’s t-test; p≤0.05 was considered to be significantly different. The predicted increase of the pY654/ß-catenin signal and the decrease of ß-catenin associated with N-cadherin were compared with one tailed Mann-Whitney test with a significance cutoff of p≤0.05. In the Barnes maze, WT and PTP1B fl/fl-Emx (KO) curves were compared by 2-way ANOVA and significance was set at p≤0.05. For strategy, a nominal logistic followed by a Pearson’s Chi-square test was used to determine differences in WT vs. KO search strategy (cued vs. non-cued). For success analysis, a nominal logistic followed by a Pearson’s Chi-square test was used to determine differences in WT vs. KO success in finding the escape box.

## Supporting Information

Figure S1 **Dendritic trees of hippocampal neurons with different PTP1B backgrounds.** (A–C) Hippocampal neurons from rat embryos were co-transfected at DIV 4 with plasmids encoding Lck-mCherry and GFP (A), GFP-PTP1B (B) or the dominant negative GFP-PTP1B(C/S) (C). At DIV10 neurons were fixed and imaged by wide-field fluorescence microscopy. Only Lck-mCherry images are shown. Note the absence of gross alterations in the overall dendritic tree by expression of wild type and C/S PTP1B. (D, E) Hippocampal neurons from WT (D) and KO (E) newborn mice were transfected at DIV4 with Lck-mCherry to visualize the neuronal morphology at DIV14. Images were taken using a fluorescence confocal microscope. Note that the overall development of dendritic trees looks similar among neurons from KO and WT mice. Scale bars, 40 µm.
(TIF)

Figure S2 **Phosphorylation of Erk1/2 in hippocampi of WT and KO mice.** Protein extracts from hippocampi of adult WT and KO mice were prepared. Western blots were first probed with a polyclonal antibody specific for phospho-p44/42 (Erk1/2), and subsequently, the membrane was stripped and re-probed with a monoclonal antibody against total Erk1/2.
(TIF)

Figure S3 **PTP1B protein levels are reduced in forebrain of PTP1Bfl/fl Emx1-cre mice.** Protein was extracted from different tissues and immunoblots were performed. Blots were stripped and reprobed for SHP-2 to control for loading. Lv: liver, Pt: pituitary, Hy: hypothalamus, Cb, Cerebellum, Hip: hippocampus, Cx: cortex.
(TIF)
