# Supplementary figures and images for: Protein Tyrosine Phosphatase PTP1B Is Involved in Hippocampal Synapse Formation and Learning
Source: PLoS One. 2012 Jul 23;7(7):e41536. doi: 10.1371/journal.pone.0041536 (PMC3402386; doi:10.1371/journal.pone.0041536)

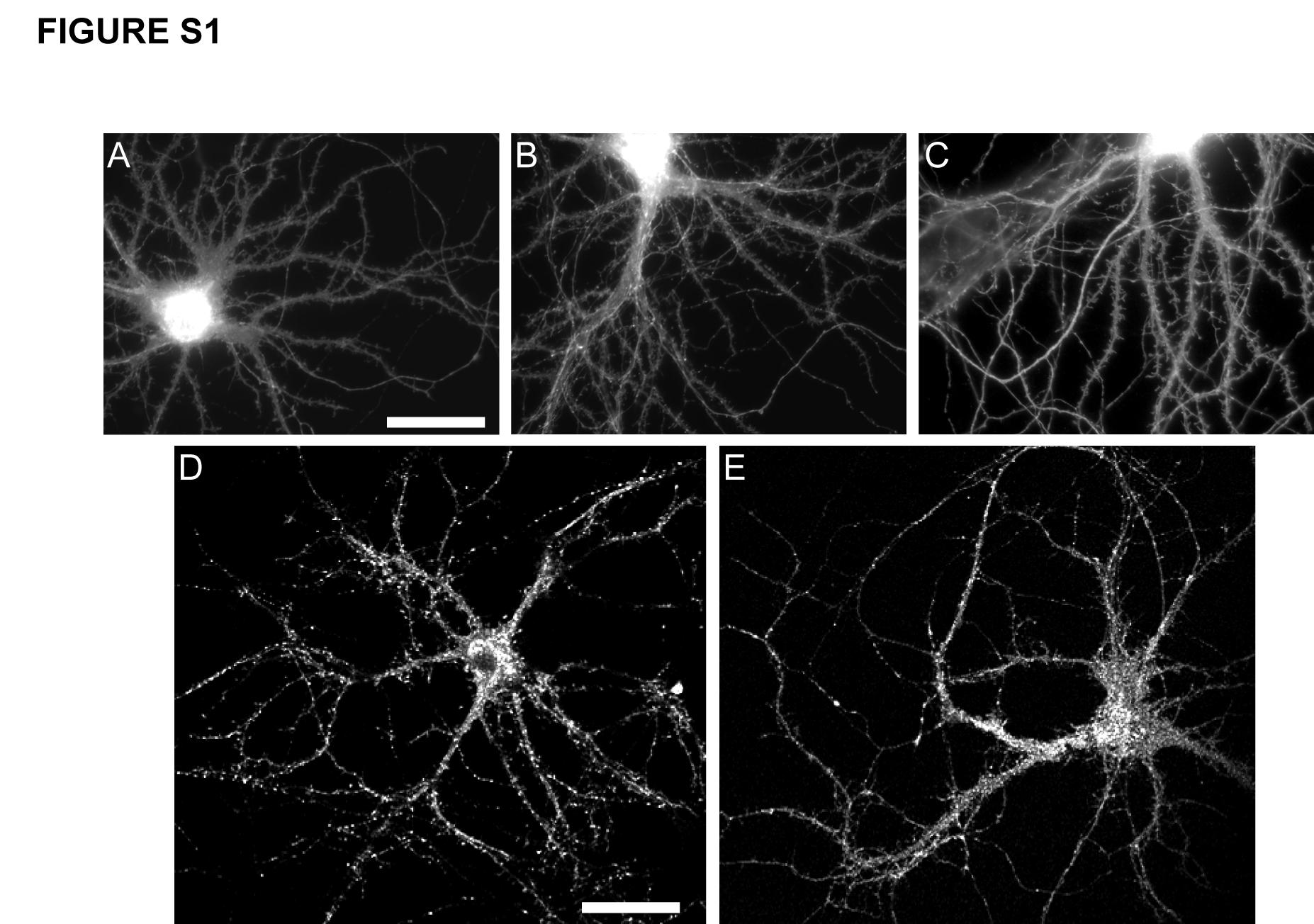

Supplement: Figure S1 — Dendritic trees of hippocampal neurons with different PTP1B backgrounds. (A–C) Hippocampal neurons from rat embryos were co-transfected at DIV 4 with plasmids encoding Lck-mCherry and GFP (A), GFP-PTP1B (B) or the dominant negative GFP-PTP1B(C/S) (C). At DIV10 neurons were fixed and imaged by wide-field fluorescence microscopy. Only Lck-mCherry images are shown. Note the absence of gross alterations in the overall dendritic tree by expression of wild type and C/S PTP1B. (D, E) Hippocampal neurons from WT (D) and KO (E) newborn mice were transfected at DIV4 with Lck-mCherry to visualize the neuronal morphology at DIV14. Images were taken using a fluorescence confocal microscope. Note that the overall development of dendritic trees looks similar among neurons from KO and WT mice. Scale bars, 40 µm. (TIF) [file pone.0041536.s001.tif]

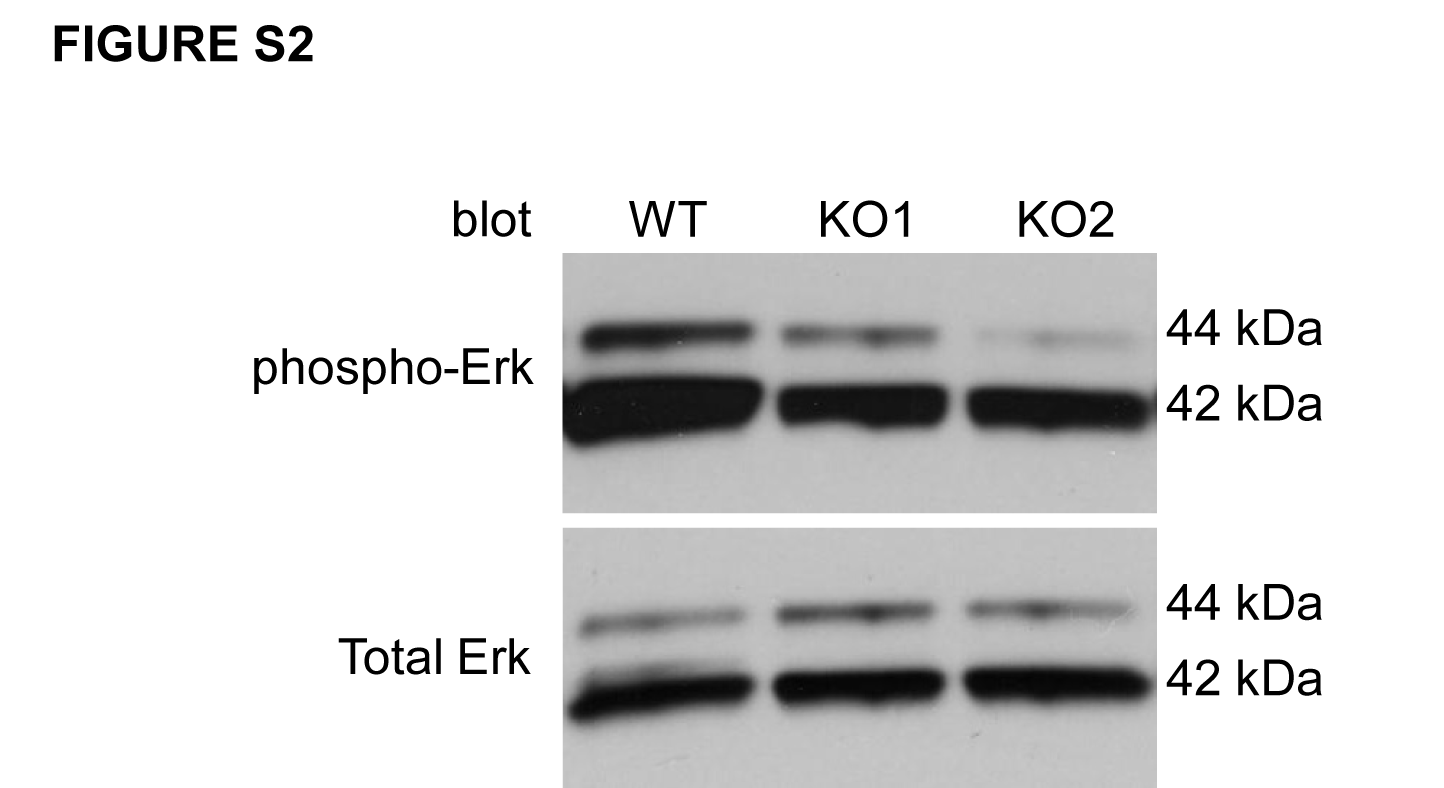

Supplement: Figure S2 — Phosphorylation of Erk1/2 in hippocampi of WT and KO mice. Protein extracts from hippocampi of adult WT and KO mice were prepared. Western blots were first probed with a polyclonal antibody specific for phospho-p44/42 (Erk1/2), and subsequently, the membrane was stripped and re-probed with a monoclonal antibody against total Erk1/2. (TIF) [file pone.0041536.s002.tif]

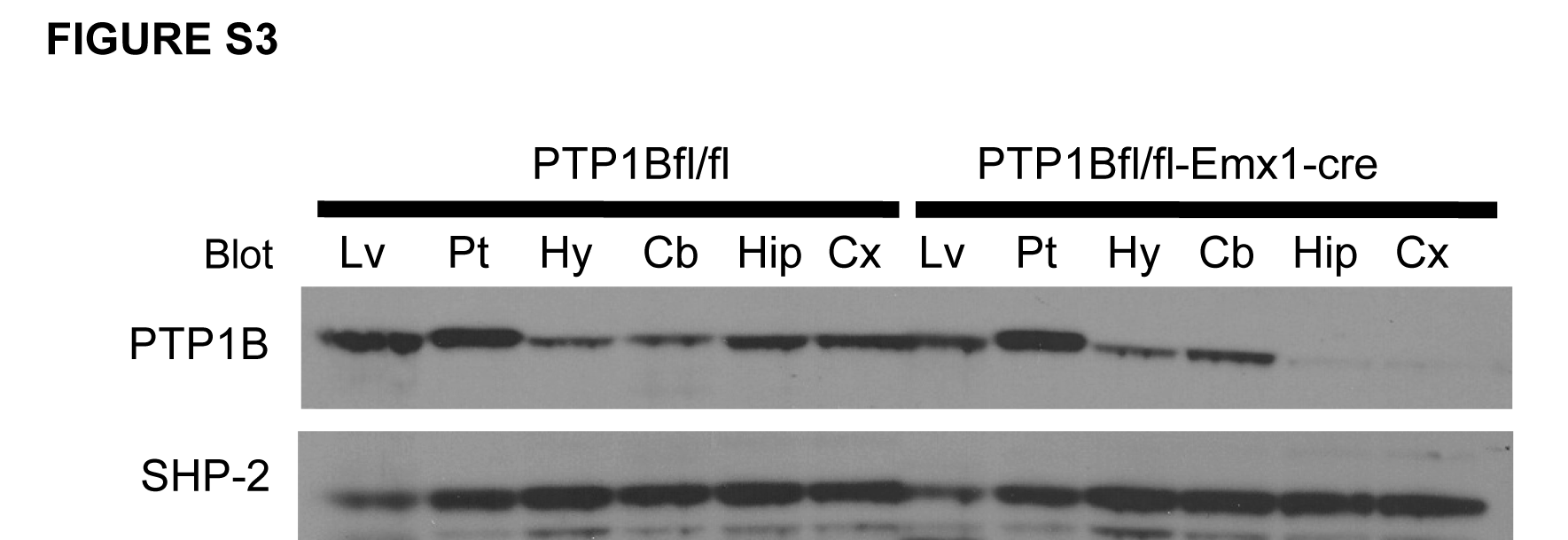

Supplement: Figure S3 — PTP1B protein levels are reduced in forebrain of PTP1Bfl/fl Emx1-cre mice. Protein was extracted from different tissues and immunoblots were performed. Blots were stripped and reprobed for SHP-2 to control for loading. Lv: liver, Pt: pituitary, Hy: hypothalamus, Cb, Cerebellum, Hip: hippocampus, Cx: cortex. (TIF) [file pone.0041536.s003.tif]
